# Default-mode and fronto-parietal network connectivity during rest distinguishes asymptomatic patients with bipolar disorder and major depressive disorder

**DOI:** 10.1038/s41398-021-01660-9

**Published:** 2021-10-23

**Authors:** Sabina Rai, Kristi R. Griffiths, Isabella A. Breukelaar, Ana R. Barreiros, Wenting Chen, Philip Boyce, Philip Hazell, Sheryl L. Foster, Gin S. Malhi, Anthony W. F. Harris, Mayuresh S. Korgaonkar

**Affiliations:** 1 Brain Dynamics Centre, Westmead Institute for Medical Research, The University of Sydney, Westmead, Sydney, NSW Australia; 2 School of Psychology, University of New South Wales, Sydney, NSW Australia; 3 Discipline of Psychiatry, Faculty of Medicine and Health, The University of Sydney, Sydney, NSW Australia; 4 Department of Radiology, Westmead Hospital, Sydney, NSW Australia; 5 Sydney School of Health Sciences, Faculty of Medicine and Health, The University of Sydney, Sydney, NSW Australia; 6 CADE Clinic, Department of Psychiatry, Royal North Shore Hospital, Sydney, NSW Australia

**Keywords:** Bipolar disorder, Scientific community

## Abstract

Bipolar disorder (BD) is commonly misdiagnosed as major depressive disorder (MDD). This is understandable, as depression often precedes mania and is otherwise indistinguishable in both. It is therefore imperative to identify neural mechanisms that can differentiate the two disorders. Interrogating resting brain neural activity may reveal core distinguishing abnormalities. We adopted an a priori approach, examining three key networks documented in previous mood disorder literature subserving executive function, salience and rumination that may differentiate euthymic BD and MDD patients. Thirty-eight patients with BD, 39 patients with MDD matched for depression severity, and 39 age-gender matched healthy controls, completed resting-state fMRI scans. Seed-based and data-driven Independent Component analyses (ICA) were implemented to examine group differences in resting-state connectivity (*p*FDR < 0.05). Seed analysis masks were target regions identified from the fronto-parietal (FPN), salience (SN) and default-mode (DMN) networks. Seed-based analyses identified significantly greater connectivity between the subgenual cingulate cortex (DMN) and right dorsolateral prefrontal cortex (FPN) in BD relative to MDD and controls. The ICA analyses also found greater connectivity between the DMN and inferior frontal gyrus, an FPN region in BD relative to MDD. There were also significant group differences across the three networks in both clinical groups relative to controls. Altered DMN–FPN functional connectivity is thought to underlie deficits in the processing, management and regulation of affective stimuli. Our results suggest that connectivity between these networks could potentially distinguish the two disorders and could be a possible trait mechanism in BD persisting even in the absence of symptoms.

## Introduction

Bipolar disorder (BD) is characterised by episodes of depressive and manic mood states. BD typically first presents in the depressed polarity and may be mistaken for major depressive disorder (MDD) [1]. Failure to recognise BD in this situation may result in prescription of antidepressants, which can increase the disease burden by causing a switch to mania, or rapid-cycling between mood states [2]. Therefore, it is imperative to identify differences in the pathophysiological mechanisms underlying BD and MDD as a step towards resolving the challenge of misdiagnosis and maximising treatment efficacy.

Resting and task-based functional imaging research has identified some of the core mechanisms underlying symptomatology of both disorders. In BD, deficits have been observed in neural circuitry associated with sensory, cognitive and attentional functioning including in the inferior parietal lobule, inferior frontal gyrus and dorsal/ventrolateral prefrontal cortices [3]. MDD has demonstrated deficits in connectivity in regions related to memory such as the hippocampus, and regions relating to negative affect and rumination such as the precuneus and posterior cingulate cortex [3]. Our group and others have previously reported differences between the two disorders based on task-related activation and connectivity of the affective circuitry [4–7]. However, task-based studies may be impacted by confounds such as levels of motivation, fatigue and disinterest. On the other hand, it has been speculated that a substantial amount of waking time is spent in a task-free ‘mind-wandering’ state where we are not consciously attending to our immediate environment [8]. Resting-state functional magnetic resonance imaging (rsfMRI) is a mode of functional imaging that examines correlations between voxels in different brain regions when the brain is in a task-free state [9]. If the time-series of voxels in two different regions correlate, these regions are assumed to be functionally connected and in communication with each other. Such functionally connected regions are considered to form an intrinsic brain network [9]. These intrinsic networks enable researchers to map the organisation of the brain and to elucidate the neuronal communication which may be contributing towards clinical pathology. rsfMRI could provide further insight into the core mechanisms that sustain the deficits observed in psychiatric disorders [10]; there is minimal level of cooperation required and because neural activity is measured during a task-free organic state there is a reduced likelihood of confounds relating to participant behaviour/disengagement.

Three key resting-state networks are consistently identified in mood disorders—the default mode network (DMN), salience network (SN) and fronto-parietal network (FPN) [3]. The DMN is related to self-referential processing and known to underlie rumination observed in these disorders whereas the SN and FPN are involved in detecting emotionally significant stimuli in one’s social milieu and attentional and cognitive executive functioning respectively. The cross-network interactions of this triple network model have been proposed to underlie the symptomatology in both mood disorders [3].

Between and within network disruptions across these networks is of interest as over-engagement of one network may result in the manifestation of one subgroup of symptoms, such as emotional instability [3], under-reliance on one network may result in another subgroup of symptoms, such as psychomotor deficits or sensorimotor dysfunction [11]. Mechanistically, in BD it could be expected that a deficit within the FPN and its interaction with SN may account for the symptoms during mania such as disorganised speech, unawareness of mania, inability to downregulate response to perceived positive stimuli, impulsivity and hyperactivity [12]. Meanwhile in MDD, abnormal interactions between the DMN and SN may result in hyper-detection and processing of perceived negative stimuli, poorer memory recall and rumination processes [13].

Previous resting-state studies in euthymic BD vs healthy controls (HC) have identified abnormalities in connections across the three networks [12, 14, 15] in particular increased connectivity between DMN and SN, and an increase in neuronal input in the FPN from limbic regions; in euthymic BD [14, 15]. These findings in a euthymic cohort suggest firstly the presence of connectivity alterations despite clinical remission which may indicate that these correlates are akin to a trait, and secondly, a possible mechanism that may underlie heightened emotional awareness observed across both polar phases (mania and depression), due to disrupted communication between these three networks [16]. A key issue in misdiagnosis of BD is that BD depression shares a similar symptom profile with unipolar depression, however it has been identified in the previous literature that BD depression differs in its neurobiology relative to unipolar depression. In resting-state studies comparing BD to MDD, depressed BD were found to have increased intra-connectivity within the FPN in comparison to MDD whereas MDD exhibited increased intra-connectivity in the DMN relative to BD [16]. Altered connectivity between the anterior insula (part of the SN) and inferior parietal lobule (part of the FPN) has also been observed in BD who are currently depressed relative to MDD [17], whilst another study also identified a pattern of inferior parietal cortex (FPN) connectivity with limbic regions differentiating BD from MDD [18]. These studies together provide a useful evidence base that this triple network model could be a likely mechanism that characterizes BD from MDD and HC, and suggest that the neurobiology of BD depression differs from unipolar depression.

However, these studies have primarily investigated symptomatic cohorts i.e. either currently depressed or currently manic. Studies that have examined euthymic BD or MDD individuals, have only compared one clinical group with HC. We currently do not know if the intrinsic connectivity differences in this triple network model between BD and MDD persist in the absence of symptoms. Investigating euthymic cohorts is important as we want to identify mechanisms that are trait-dependent as opposed to state-dependent. This is because trait markers can provide insight into the potential biological factors that predispose an individual towards a particular disorder and can allow researchers to investigate the disease mechanisms in order to generate improved diagnostic methods and treatment [19]. State markers are dynamic in comparison and are indicative of the current symptom status in patients [19]. Whilst identifying changes in symptoms along the trajectory of a disorder is key i.e., the development of depressive symptoms in BD preceding mania; identifying trait markers is fundamental to understanding the neurobiological changes that trigger the onset of symptoms. The current study aims to investigate the gap of examining resting state connectivity in asymptomatic BD and MDD cohorts in order to identify pathophysiological mechanisms that may be able to discriminate between these groups. We hypothesised that the BD group would be expected to exhibit increased neural connectivity in the FPN and SN in both an inter-network and intra-network manner relative to MDD and controls, whereas the MDD group would be expected to elicit increased inter-and intra network connectivity in the DMN relative to BD and controls.

## Methods and materials

### Participants

120 Participants recruited for this study were between the ages of 18–65 and included 39 euthymic MDD participants, 40 euthymic BD participants and 41 HC. The sample size for each group was based a pilot data analysis that provided at least 95% power to observe similar effect size for functional connectivity analyses. The sample size are also larger than previously published studies. Further information on the demographics of the participants is found in Table 1. Testing took place at the Westmead Institute for Medical Research. BD and MDD participants were recruited through various methods, including community flyers and clinician referrals from the Western Sydney Local Health District. Both BD and MDD groups met the DSM-V diagnostic criteria for BD type-I or MDD, as well as being in remission at the time of testing. The period of remission was characterised by at least 14 days of scoring less than 7 on the Hamilton Depression Rating Scale (HDRS [20], as well as not meeting criteria for (hypo)manic episodes, according to the Young Mania Rating Scale (YMRS) [21]. To control for the possibility that our MDD group could potentially progress to BD, we calculated the average duration of illness in the MDD group. From this we found that the average length of illness in the MDD group was 15 years, which is beyond the average duration that BD manifests following a depressive episode [2].

**Table 1:** The demographics and clinical characteristics of all participants.

	BD (*n* = 38)	MDD (*n* = 39)	HC (*n* = 39)	*F/X*^*2*^ value	*p-*value
Age (Mean ± SD)	39.15 ± 13.75	36.20 ± 11.81	36.97 ± 13.67	0.524	0.71
Sex, % Female	63.00	56.00	53.00	0.357	0.63
Hamilton Depression Rating Scale, (Mean ± SD)	4.32 ± 3.40*	5.11 ± 3.72*	1.61 ± 1.98	13.204	<0.001
Young Mania Rating Scale, (Mean ± SD)	2.24 ± 3.75	1.39 ± 2.91	0.717 ± 1.71	2.632	0.07
No. of Depressive Episodes, (Mean ± SD)	6.80 ± 6.10	7.16 ± 6.83	–	0.031	0.82
DASS Depression, (Mean ± SD)	2.76 ± 4.00*	2.60 ± 3.20*	0.763 ± 1.17	5.050	0.04
DASS Anxiety, (Mean ± SD)	3.23 ± 4.04*	3.25 ± 4.35*	1.50 ± 1.88	2.981	0.01
DASS Stress, (Mean ± SD)	2.76 ± 3.44*	2.85 ± 3.37*	1.15 ± 1.55	4.030	0.01
Mean Framewise Displacement, (Mean ± SD)	0.17 ± .075	0.14 ± 0.057	0.14 ± 0.049	3.013	0.06
Lithium, *n (*%)	11 (28)	–	–	–	–
Antipsychotic, *n (%)*	11 (28)	2 (5)	–	–	–
Anticonvulsant, *n (%)*	8 (20)	–	–	–	–
SSRI *n (%)*	3 (8)	8 (21)	–	–	–
SNRI, *n (%)*	1 (3)	3 (7)	–	–	–

*DASS* Depression Anxiety Stress Scale, *SD* Standard Deviation, *SSRI* Selective Serotonin Reuptake Inhibitor, *SNRI* Selective Norepinephrine Reuptake Inhibitor.

*indicates significantly different from controls at *p* < 0.05. There were no differences between BD & MDD groups.

Following expression of interest in the study and consent, BD and MDD participants were assessed for eligibility. Participants completed the Structured Clinical Interview for DSM-V (SCID-5), HDRS, YMRS and the Depression Anxiety Stress Scales (DASS) [22]. HC were recruited through the community and were assessed using the Mini-International Neuropsychiatric Interview to ensure that they were free of any psychiatric illness. All participants were required to be in good physical health, have no past or current substance dependence, any history of a traumatic brain injury or any contraindication that would prevent them from partaking in the MRI scan.

Of the 120 participants, 2 HC and 2 BD were excluded for excessive movement during the fMRI scan, no MDD were excluded from the analyses, resulting in data from 116 participants remaining for analyses (38 BD, 39 MDD and 39 HC). Participants gave informed consent to participate in the study and were given the option to withdraw from the study at any time. This study was conducted in accordance with the ethical guidelines of the institutional review board (Western Sydney Local Health District human research ethics committee) and in accordance with the Declaration of Helsinki.

### fMRI acquisition

A 3 T Siemens PRISMA scanner (Siemens Healthcare, Erlangen, Germany) was used to acquire MRI data at the Department of Radiology in the Westmead Hospital, using a 64-channel head and neck array coil. Functional MRI data was acquired using echo-planar imaging (repetition time/echo time = 1500 ms/33.0 ms. field of view = 255 mm, isotropic voxel size = 2.5 × 2.5 × 2.5 mm^3^, phase encoding direction = A ≫ P, GRAPPA = 2, 60 slices at 2.5 mm thickness covering the whole brain). The MRI scan was comprised of the resting state scan which was first in the sequence and involved the participant focusing on a fixation cross for 8 minutes 12 sec, (only the resting state MRI scan is analysed and discussed in this article, further details of the full fMRI protocol can be found in a previous study by our research group [5]. Structural MRI data was acquired through a 3D high-resolution T1-weighted gradient echo sequence with repetition time/echo time = 2400 ms/2.21 ms; phase encoding direction = A ≫ P, GRAPPA = 2, inversion time = 900 ms, field of view = 256 mm, flip angle = 8°, 192 sagittal slices covering the whole brain with isotropic voxel size of 0.9 mm^3^ and an acquisition time of 6 minutes 23 sec. The T1-weighted scan was used as the standard reference image for normalisation in the pre-processing steps.

### fMRI data preprocessing

Neuroimaging data was pre-processed using the CONN functional connectivity toolbox (Version 18b) [23] and SPM12 (http://www.fil.oin.ucl.ac.uk/spm). The images underwent realignment and unwarping by co-registering all scans to a standard reference image, which was the first volume. Slice-timing correction was applied to correct any temporal misalignment that occurred during the scan acquisition period. The Artifact Detection Tools scrubbing procedure was used to screen for potential motion outliers in the data. Scans exhibiting framewise displacement above 0.9 mm were flagged as outliers. Our exclusion criteria for motion were determined on the number of volumes flagged as outliers divided by the total number of volumes in each individual scan (320 volumes); any participants with >25% of outlier volumes were excluded from the analysis (see Table S1 of supplement). The data was normalised and segmented into the appropriate tissue class (Grey matter/white matter/CSF) using the mean BOLD signal as a reference image for the functional data and the T1-weighted scan as the reference for the structural data. Following normalisation, the data was smoothed using a Gaussian kernel of 6 mm full width half maximum. The denoising step in CONN used CompCor which extracts potential noise components from the white matter and cerebrospinal regions, subject-motion parameters and scrubbing procedure to correct for noise-related signal [23]

### Neuroimaging data analysis

The data was analysed using the CONN functional connectivity toolbox (Version 18b) [23]. Two analysis techniques were applied to the dataset to investigate connectivity differences between BD and MDD. The first analysis was seed-based where 14 region of interest (ROI) masks was selected to cover the three neural networks outlined in our hypotheses (see Figure S1 of supplement). ROIs were defined using spheres 8–10 mm in radius (depending on brain region) or selected from the MARSeille Boîte À Région d’Intérêt (MarsBaR) toolbox. For the SN the selected masks were bilateral anterior insula (MarsBaR), bilateral Amygdala (MarsBaR), the dorsal anterior cingulate cortex (dACC) (centred around MNI co-ordinates 0, 24, 38). For the FPN the selected masks were: the dACC as specified previously, right dorsolateral PFC (RDLPFC) (51, 15, 48), left DLPFC (−36, 15, 57), left anterior inferior parietal lobule (−38, −52, 40), right anterior inferior parietal lobule (38, −50, 42), the left superior parietal lobule (−28, −60, 44) and the right superior parietal lobule (32, −60, 42). For the DMN, the selected masks were the medial PFC (mPFC) (−1, 45, 5), the subgenual ACC (sgACC) (0, 24, −8), the bilateral precuneus (MarsBaR) and the posterior cingulate cortex (PCC) (−6, 52, 40). The seed-based analysis analysed functional connectivity differences between groups using a 14×14 matrix of all viable connections between all ROIs. Multiple comparisons were controlled for using false discovery rate method with a corrected threshold of *p* < 0.05.

The second analysis was independent-component analysis (ICA), adopting a data-driven approach to the a priori approach of seed-based ROI analysis. Prior to ICA, the number of components within the dataset were estimated using the Group ICA of fMRI Toolbox (GIFT) v3.0b. The components were first estimated for each subject using minimum description length criteria before using the mean of the individual subject results to estimate the average number of components across the dataset. Using the default settings for CONN 18b, the group-ICA analysis was run using GICA3 back-projection and G1 FastICA with dimensionality reduction of 64 and the number of independent components (IC) was set to 32 based on component estimation. Subsequently, the 32 ICs were matched to a spatial template of neural networks provided by the CONN functional network atlas(see Figure S2 of supplement) and correlation co-efficient values indicated which network regions were predominant in each component. Group comparisons were performed to evaluate connectivity differences related to each ICN. The statistical threshold was set to voxel-wise *p* < 0.001 at uncorrected level to define the voxel size, then a cluster-wise correction was applied at a threshold of false discovery rate *p* < 0.05 to determine significant clusters.

### Medication effects

We conducted *t*-tests for the BD and MDD groups to determine if concurrent medication influenced neural measures. Further information on the results are reported in Tables S2 and S3 of the supplement.

### Correlation with clinical measures

Fisher’s Z scores for significant neural connections in the seed-based analyses between BD and MDD were extracted using the CONN toolbox and input into the Statistical Package for the Social Sciences (SPSS) (Version 22; IBM Corp., Armonk, NY); to examine if there were significant correlations between the connectivity of the ROIs and clinical measures such as the HDRS, DASS, YMRS and previous depressive episodes. Pearson’s partial correlations were used, controlling for diagnostic group to investigate any correlations between neural and clinical measures independent of diagnosis. Secondary to this analysis, we conducted a within-group bivariate correlational analysis to determine if diagnostic category was a factor in the correlations between neural and clinical measures. These analyses were similarly replicated for the significant clusters identified in the ICA analysis. As these were exploratory secondary analyses an uncorrected *p* < 0.05 value was used.

## Results

### Demographics

Table 1 illustrates demographic and clinical characteristics data for BD, HC and MDD. BD and MDD were gender and age matched to the HCs. There were group differences for the HDRS and DASS scales but subsequent post-hoc tests indicated that the differences were only between BD vs HC and MDD vs HC, the two clinical groups were not different from each other.

### Resting state seed-based functional connectivity differences within the DMN-FPN-SN model between BD and MDD

**Fig. 1:**
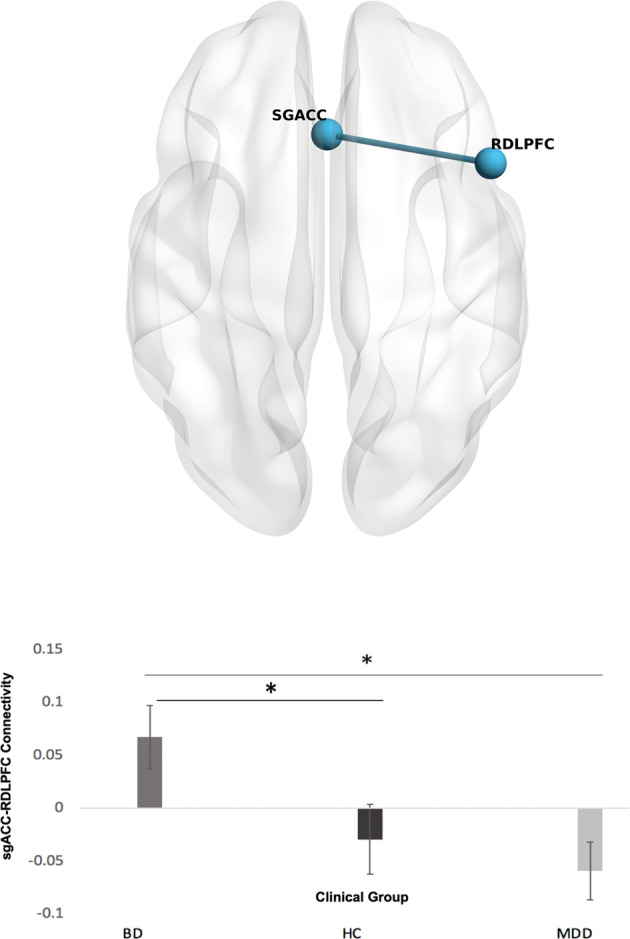
Connectivity differences between BD and MDD patient groups, and control group in seed-based analyses. The results from the seed-based analyses indicated significantly greater connectivity between the sgACC-RDLPFC during rest, in the BD group relative to MDD and controls. The graph below illustrates the differences in connectivity for the sgACC-RDLPFC across the three groups. *p < 0.05.

As illustrated in Fig. 1, after adjusting for multiple comparisons the BD group was found to exhibit a significant level of increased functional connectivity between the sgACC and RDLPFC (*t* (113) = 2.95, FDR *p* = 0.049) during rest, relative to MDD. The BD group also had greater connectivity than HC (*p* = 0.026) but there were no connectivity differences between MDD and HC.

### Resting state ICA differences between BD and MDD

**Fig. 2:**
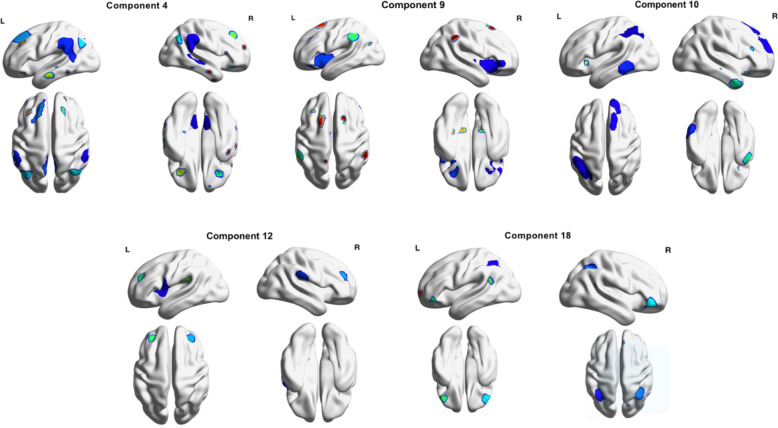
The components in the dataset which correspond to the three networks of interest. IC 4 consisted of the posterior DMN regions and also anterior regions such as the anterior cingulate gyrus and frontal pole. IC 9 was comprised of regions relating to the SN such as the paracingulate gyrus, insular cortex and frontal orbital cortex. IC 10 consisted of regions relating to the FPN such as the frontal pole, middle frontal gyrus, middle temporal gyrus and other regions such as the cerebellum. IC 12 was comprised of SN regions such as the insular cortex and other regions such as the frontal operculum cortex and precentral gyrus. IC 18 consisted mainly of regions that are specific to the posterior portion of the DMN such as the posterior cingulate gyrus and precuneus.

**Fig. 3:**
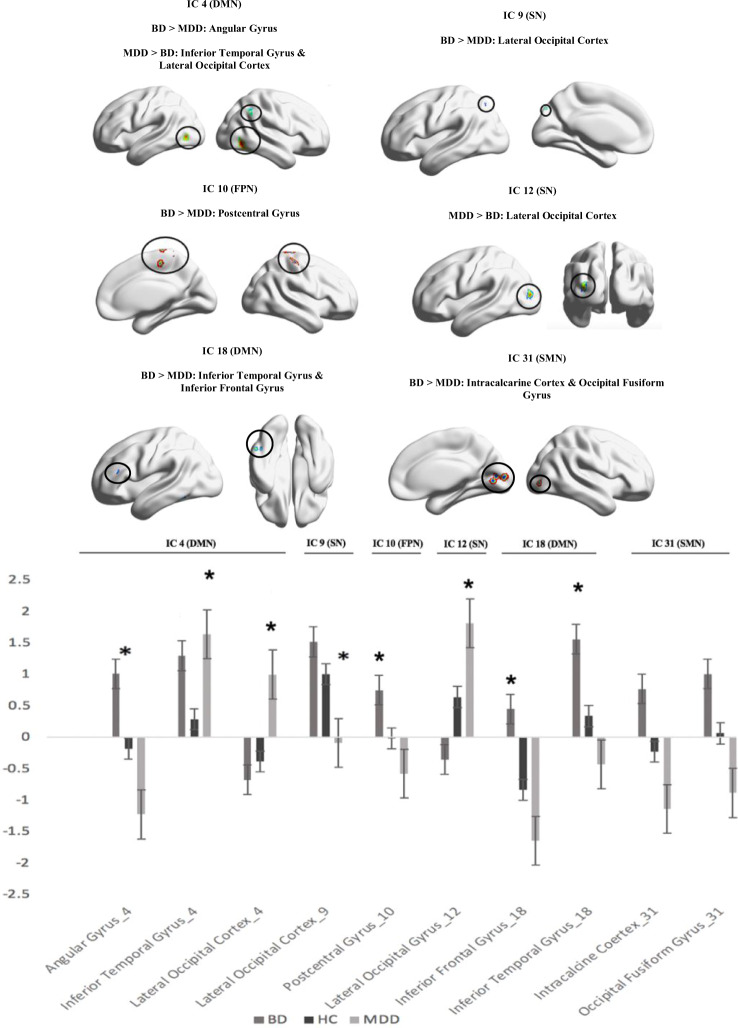
ICs with significantly different connectivity profiles between BD and MDD. The BD group exhibited greater connectivity than MDD in the following components: 1. IC 4 (DMN) with the angular gyrus, 2. IC 9 (SN) with the lateral occipital cortex, 3. IC 10 (FPN) with the postcentral gyrus, 4.. IC 18 (DMN) with the inferior temporal gyrus and inferior frontal gyrus and 5. IC 31 (Sensorimotor Network) with the intracalcarine cortex and occipital fusiform gyrus. On the other hand, MDD exhibited greater connectivity than BD in the following components: 1. IC 4 (DMN) with, lateral occipital cortex and inferior temporal gyrus and 2. IC 12 (SN) with the lateral occipital cortex. *p < 0.05.

Figure 2 illustrate the ICs that matched to the three brain networks. Table 2 and Fig. 3 illustrate differences in connectivity between BD and MDD. The BD group exhibited greater connectivity than MDD in the following components:1. IC 4 (DMN) with the angular gyrus (*t* (113) = 5.42, FDR *p* < 0.001), 2. IC 9 (SN) with the lateral occipital cortex (*t* (113) = 4.22, FDR *p* < 0.005), 3. IC 10 (FPN) with postcentral gyrus (*t* (113) = 4.31, FDR *p* < 0.001), 4. IC 18 (DMN) with the inferior temporal gyrus (*t* (113) = 4.58 FDR *p* < 0.026) and inferior frontal gyrus (*t (*113) = 4.26, FDR *p* < 0.046) and 5. IC 31 (SMN) with the intracalcarine cortex (*t* (113) = 4.10, FDR *p* < 0.048) and occipital fusiform gyrus (*t* (113) = 4.04, FDR p < 0.048). On the other hand, MDD exhibited greater connectivity than BD in the following components: 1. IC 4 (DMN) with the lateral occipital cortex (*t* (113) = 4.24, FDR *p* < 0.008) and inferior temporal gyrus (*t* (113) = 5.42, FDR *p* < 0.001) and 2. IC 12 (SN) with the lateral occipital cortex (*t* (113) =4.45, FDR *p* < 0.001), There were connectivity differences for both BD vs HC and MDD vs HC which are summarised in Tables S4 and S5 of the supplement. In the clusters that were significant for patient group vs HC, connectivity appeared to be lower for HC.

**Table 2:** Differences in ICA network connectivity between BD & MDD.

Component	Neural network	Contrast	Regions	MNI Space (x y z)	Voxels	T value	FDR *p*-value (<0.05)
9	SN	BD > MDD	Lateral Occipital Cortex	−18 −70 50	114	4.22	0.005
18	DMN	BD > MDD	Inferior Temporal Gyrus	−52 −54 −18	133	4.58	0.026
			Inferior Frontal Gyrus	−42 30 10	51	4.26	0.046
10	FPN	BD > MDD	Postcentral Gyrus	12 −36 72	167	4.31	0.001
4	DMN	BD > MDD	Angular Gyrus	42 −56 58	142	5.42	0.001
31	SMN	BD > MDD	Intracalcarine Cortex	4 −74 2	63	4.10	0.048
			Occipital Fusiform Gyrus	36 −76 6	50	4.04	0.048
4	DMN	MDD > BD	Inferior Temporal Gyrus	46 −56 −4	81	5.42	0.008
4	DMN	MDD > BD	Lateral Occipital Cortex	−38 −62 −4	74	4.24	0.001
12	SN	MDD > BD	Lateral Occipital Cortex	−38 −88 10	149	4.45	0.001

Effects significant at corrected false discovery rate (*p* < 0.05) are shown. *ICA* Independent Component Analysis, *BD* Bipolar Disorder, *MDD* Major Depressive Disorder, *DMN* Default Mode Network, *SN* Salience Network, *FPN* Frontoparietal Network, *SMN* Sensorimotor Network.

### Correlations between neural and clinical measures between BD and MDD and medication analysis

There were no significant correlations between sgACC-RDLPFC connectivity and clinical measures across both BD and MDD patients or across the pooled cohort after controlling for diagnostic category. Similarly, there were no significant correlations for the ICA connectivity clusters and clinical measures. T-tests were conducted to measure potential within-groups effects of medication on neural measures, the results were insignificant except for the MDD SSRI group and FPN-postcentral gyrus connectivity (found in Table S3 of supplement). An analysis of covariance was also conducted to investigate between-group effects of medication on neural measures and returned insignificant results. The connectivity results remained significant after controlling for medication.

## Discussion

The aim of this study was to investigate resting state connectivity in the FPN, SN and DMN in euthymic BD and MDD populations, to identify mechanisms that distinguish the two disorders. As hypothesised, there were differences in connectivity between the three networks between the patient groups. Specifically, seed-based analyses indicated that the BD group displayed significantly greater resting connectivity between the RDLPFC, a region that is part of the FPN, and the sgACC, a region that is part of the DMN, relative to MDD and HC. The ICA results found greater connectivity for the BD group related to all of the three networks and sensorimotor network and particularly between the FPN and the DMN as observed for the seed-based analysis. We also observed greater connectivity for the MDD group compared to BD, between the DMN and SN to the visual regions.

The sgACC is part of the DMN and is involved in the processing of internalised emotive stimuli [24]. The DLPFC is part of the FPN which plays a role in inhibitory/attentional allocation processes [3]. Past studies in task-based and resting state fMRI have found similar altered connectivity patterns between the sgACC and RDLPFC, and DMN and FPN, specifically in euthymic BD. For example, Favre et al. [25] identified that euthymic BD had increased connectivity between nodes of the DMN and FPN relative to HC, suggesting altered functioning between the two networks and indicating that the same pattern has been observed during task processing [24]. Again, a different study identified greater resting connectivity between the sgACC/medial prefrontal cortex and RDLPFC in euthymic BD versus HC, which replicates our findings [25].

Our finding of greater connectivity of the sgACC to the DLPFC in BD during rest could be a possible mechanism for the emotional disturbances that are experienced during episodes. For example, the higher level of emotional awareness that accompanies the depressive bipolarity, and the lack of self -awareness which accompanies a manic episode. The sgACC has also been implicated in MDD literature particularly the work of Helen Mayberg (Mayberg et al. [26]), and has also been the primary target for transcranial magnetic stimulation and deep-brain stimulation treatments in MDD [27]. However, a key aspect to note is that increased sgACC metabolism normalises in MDD patients who respond to treatment and that increased sgACC metabolism is also associated with a higher severity of depression such as treatment-resistant depression [28]. The MDD cohort in our study were treatment-responsive which may explain the lack of differences in this connectivity relative to controls. Previous work from our lab using this cohort also identified increased right amygdala activation but not increased sgACC activation [5].

Our previous study using an emotional regulation task in the same cohort also found sgACC differences between the two cohorts. However, failed to demonstrate functional connectivity related to the sgACC during both emotion regulation and passive processing to differentiate the two patient groups [5]. This could suggest this feature could be specifically related to resting state and reflect an intrinsic connectivity difference between the two cohorts. Also, functional activation of the DLPFC is found to be altered in both BD([25]) and MDD [29]. Treatment studies using repetitive transcranial magnetic stimulation (rTMS)targeting the DLPFC in both MDD and BD have been associated with improvement in depressive and cognitive symptoms [30, 31]. Research has shown that the mechanism for rTMS treatment is via the connectivity of the DLPFC to the sgACC [31]. Importantly, rTMS targeting the DLPFC in BD was found to alleviate both depressive and manic symptoms, which may suggest a differential role of this connectivity feature between BD and MDD [32, 33].

The first notable finding from the ICA analyses, which is similar to the seed-based analysis finding, is the greater connectivity between the DMN and inferior frontal gyrus (DLPFC), an FPN node in BD, relative to MDD and HC. The inferior frontal gyrus is related to cognitive/emotional control and in past literature has been found to be underactive in both symptomatic and euthymic BD patients relative to HC and has been associated as a trait mechanism in at-risk cohorts [34, 35]. Connectivity from the DMN to the inferior frontal gyrus may indicate disrupted functioning of the FPN which could relate to the difficulties observed in appraising and subsequently regulating emotionally significant stimuli. This region has also been reported as an endophenotype of BD [34]. Its role in BD is also supported in our work as the significant difference in DMN-inferior frontal gyrus connectivity was present for BD vs HC and MDD but not in MDD vs HC in our analysis (found in Table S4 and S5 of the supplement).

The second finding is that both clinical groups (BD relative to MDD only and MDD relative to HC) exhibited differences in SN and lateral occipital cortex connectivity. As salience of stimuli is often detected visually this connectivity pattern may relate to hypervigilance and sensitivity to emotional stimuli that is a feature of mood disorders and particularly depression. This finding supports the role of the SN in mood disorders. A key hub of the SN is the anterior insula and is thought to account for switching between the executive networks and DMN [36]. Alterations in the anterior insula have been documented across BD and MDD and it is possible that it may account for hypoactivity of executive network functioning and hyperactivity of the DMN and sensory networks. This alteration may further exacerbate mood states and clinical symptoms by enhancing rumination and inability to reorient attention from emotionally significant stimuli (Menon et al, [3, 17, 37]). Specifically with our finding, as salient stimuli is often detected visually this may explain why connectivity between the SN and visual regions was greater in BD and MDD relative to controls.

Thirdly, there was a difference in connectivity between BD and MDD for the SMN with visual occipital regions (BD > MDD), FPN and sensory regions such as the postcentral gyrus (BD > MDD), and the DMN and angular gyrus (BD > MDD) and lateral occipital cortex (MDD > BD). We could speculate that this could be a likely mechanism that underlies difficulty integrating sensory and affective processes in BD relative to HC and MDD, and could underpin disorganised speech, psychomotor deficits and mania that is core to the illness of BD [11]. As we used a DMN-SN-FPN network model in our hypotheses, this finding of the SMN was not hypothesised and was unexpected, and could suggest that this network is relevant when considering pathophysiology of mood disorders.

The following limitations should be considered. While we interpreted our findings in light of known functional associations of these regions and networks, it is important to note that our study did not directly evaluate any associations with cognitive or emotional symptoms directly or used task based fMRI. Hence these interpretations should be treated with caution and tested in future work with symptom data or task based fMRI. It is also important to note that the neural features we observed may also be inter-episodic signatures that are helping to sustain remission.

A potential limitation of our study may be including currently medicated participants. Whilst it was our aim to investigate differences in a euthymic cohort, medication may have impacted neural activity. Lithium may influence muscle physiology thus potentially affecting the neurovascular relationship that underpins the BOLD technique in fMRI [38]. However, this effect of lithium has been noted as occurring globally across the brain rather than targeting specific areas of the brain [39]. To control for these potential neurochemical factors, we also carried out an analysis (see Results section) which returned insignificant results for a medication effect on neural activity, with the exception of MDD-SSRI medicated group who returned a significant medication effect on connectivity between the FPN and the postcentral gyrus; however, considering the small sample size of this group (*n* = 6) these results should be interpreted with caution. Another limitation is that we did not include a follow-up to determine if the patients in the MDD cohort had remained as euthymic MDD patients or progressed to BD. The mechanisms between these instances of chronic MDD and MDD transforming into BD vary and require further investigation. An optimal design to address this limitation may include analysing the resting state scans to determine if there are any possible neural factors in the MDD group that may lead towards BD conversion. The findings in the BD group of our study may represent a “scar” from repeated episodes of significant intensity and illness length; thus investigating potential antecedents from MDD to BD conversion is important. In our study however, the conversion of MDD to BD is unlikely, given that the duration of MDD illness and the age range of our cohort. Finally, whilst using a euthymic cohort allowed us to investigate trait markers, we did not test our identified markers in depressed and manic BD patients; if a marker is trait-specific it should appear across all mood states of the disorder and also possibly in individuals at genetic risk for developing BD. Validating the mechanisms obtained in our euthymic BD cohort against symptomatic BD patients and at-risk individuals warrants further investigation.

In summary, this study aimed to identify neural mechanisms during resting state, particularly in networks underlying rumination, salience, cognitive processing that could discriminate BD from MDD. Our findings demonstrate DMN-FPN connectivity was a key distinguishing feature of BD relative to MDD. This DMN-FPN connectivity finding unique to BD may be core to the illness and akin to a trait mechanism not impacted by mood states. Future work should explore this neural mechanism in individuals with genetic risk for this disorder. A possible avenue for further research could also be to use the sgACC-RDLPFC connectivity feature to study treatment response mechanisms in symptomatic BD using brain stimulation techniques.

## Supplementary information

Supplement

## Data Availability

Data would be made available upon reasonable request to the corresponding authors.
