# Supplementary material for: Default-mode and fronto-parietal network connectivity during rest distinguishes asymptomatic patients with bipolar disorder and major depressive disorder
Source: Transl Psychiatry. 2021 Oct 23;11:547. doi: 10.1038/s41398-021-01660-9 (PMC8542033; doi:10.1038/s41398-021-01660-9)
Supplement: Supplementary file 1 — Supplement [file 41398_2021_1660_MOESM1_ESM.docx]

***Supplementary information***

**Default-Mode and Fronto-Parietal Network Connectivity during Rest Distinguishes Asymptomatic Patients with Bipolar Disorder and Major Depressive Disorder**

*Sabina Rai ^1^, Kristi R. Griffiths ^1^ , Isabella A. Breukelaar ^1,6^, Ana R. Barreiros ^1^, Wenting Chen^1^, Philip Boyce^2^, Philip Hazell^2^, Sheryl L. Foster^3,5^, Gin S. Malhi^2,4^, Anthony W.F. Harris^1,2^, Mayuresh S. Korgaonkar^1,2, 5^*

^1^Brain Dynamics Centre, Westmead Institute for Medical Research, The University of Sydney, Westmead, Sydney, Australia

^2^Discipline of Psychiatry, Faculty of Medicine and Health, The University of Sydney, Sydney, Australia

^3^Department of Radiology, Westmead Hospital, Westmead, New South Wales, Australia

^4^CADE Clinic, Department of Psychiatry, Royal North Shore Hospital, Sydney, NSW, Australia

^5^ Sydney School of Health Sciences, Faculty of Medicine and Health, The University of Sydney, Sydney, Australia

^6^ School of Psychology, University of New South Wales, Sydney, Australia

**S1. Selecting ROI seed regions for the triple network model**

To satisfy the criteria of our hypotheses, we needed to select ROIs from each of the three neural networks - salience network (SN), the default mode network (DMN) and frontoparietal network (FPN). The target seed regions were identified from meta analyses. We used: Williams LM. Precision psychiatry: a neural circuit taxonomy for depression and anxiety. Lancet Psychiatry. 2016 May;3(5):472-80. doi: 10.1016/S2215-0366(15)00579-9. Epub 2016 Apr 14. PMID: 27150382; PMCID: PMC4922884 to define the networks and their regions, and we used meta analyses for the FPN/CCN (Niendam, T.A., Laird, A.R., Ray, K.L., Dean, Y.M., Glahn, D.C. and Carter, C.S., 2012. Meta-analytic evidence for a superordinate cognitive control network subserving diverse executive functions. *Cognitive, Affective, & Behavioral Neuroscience*, *12*(2), pp.241-268),

DMN (Fox, M.D., Snyder, A.Z., Vincent, J.L., Corbetta, M., Van Essen, D.C. and Raichle, M.E., 2005. The human brain is intrinsically organized into dynamic, anticorrelated functional networks. *Proceedings of the National Academy of Sciences*, *102*(27), pp.9673-9678),

And SN (Menon, V., 2011. Large-scale brain networks and psychopathology: a unifying triple network model. *Trends in cognitive sciences*, *15*(10), pp.483-506.) Neural networks are summarised below in Figure S1.

**
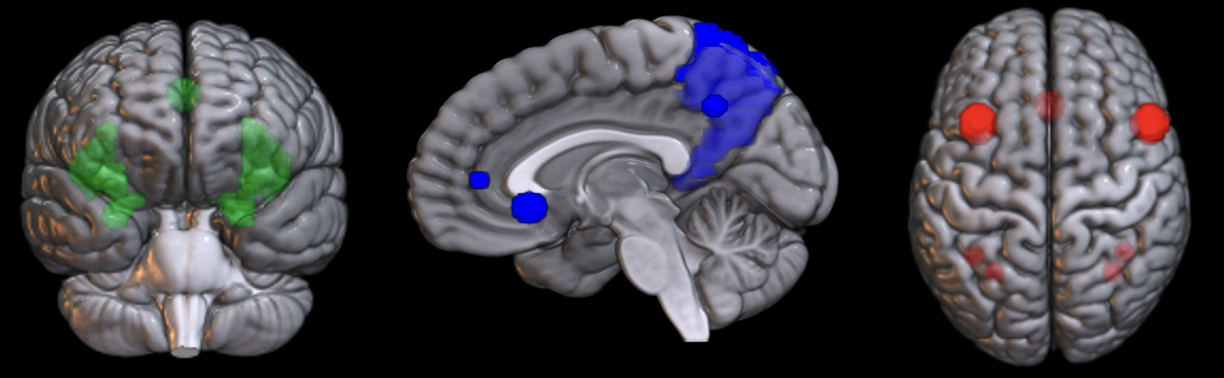
**

**Figure S1.** The tripartite neural networks used in the seed-based ROI analysis. The left image depicts the SN and is comprised of the dACC, sgACC, bilateral amygdala and bilateral anterior insula. The middle image illustrates the DMN and is comprised of the mPFC, the PCC, precuneus and the sgACC. The right image depicts the FPN and is comprised of the bilateral anterior IPL, bilateral SPL, dACC and bilateral dlPFC.

**S2. Controlling for motion and framewise displacement between groups**

In CONN, the default setting for framewise displacement sets < 0.9mm as the boundary for acquisitions to not be flagged as potential outliers, any acquisitions beyond this limit are flagged. To determine average framewise displacement between groups, the raw values of the CONN generated first-level covariate ‘QC_timeseries’ were averaged and not aggregated, to obtain an average framewise displacement value for each participant. Following this the values were exported to SPSS where the average and standard deviations were computed for the three groups as well as a one-way ANOVA to determine if motion was a significant factor between groups. The ANOVA returned insignificant results as illustrated below in Table S1.

**Table S1.** One-Way ANOVA between groups for Mean Framewise Displacement.

| **Contrast** | **Direction** | ***F* score** | ***p* value** | |  |
| --- | --- | --- | --- | --- | --- |
| **Mean Framewise Displacement** | Between Groups | 3.013 | | .053 | |

**S3.** **Analysing the potential impact of medication on sgACC-RDLPFC finding and ICA findings.**

Analysing the impact of medication on neural measures across the two diagnostic categories was not possible as the BD and MDD were on different medications, lithium and selective serotonin reuptake inhibitors (SSRIs) respectively. Thus, we decided to investigate potential medication effects within each group independently. As we did not have blood serum samples we were not able to examine lithium metabolism/concentration, instead we separated each clinical group into current medicated vs non-current medicated and compared the corresponding neural measures using independent sample t-tests. For example, those in the BD group that were currently on lithium (*n* = 11) were compared with those in the BD group that were currently not medicated on lithium (*n* = 29), similarly to how those in the MDD group that were currently taking SSRIs (*n* = 6) were compared with those in the MDD group that were not currently taking SSRIs (*n* = 33). Due to excess motion in the fMRI scan two of the BD cohort were removed from the analysis leaving (*n* = 9) remaining for the medication analysis. The results are summarised below in Table S2 for sgACC-RDLPFC and Table S3 for the ICA findings. There appeared to be no significant impact of medication in any of the clinical groups.

**Table S2.** The effect of medication on sgACC – RDLPFC finding in the MDD and BD group (MDD - Major Depressive Disorder, BD – Bipolar Disorder, SSRI - Selective Serotonin Reuptake Inhibitors, sgACC - Subgenual Anterior Cingulate Cortex; RDLPFC – Right Dorsolateral Prefrontal Cortex).

| Patient Group |  | Region | *P* Value | *T* Score | |  | |  |
| --- | --- | --- | --- | --- | --- | --- | --- | --- |
| MDD SSRI vs MDD not currently on SSRI |  | sgACC - RDLPFC | .473 | | .725 | |  | |
| BD Lithium vs BD not currently on Lithium |  | sgACC -RDLPFC | .346 | | .954 | |  | |

**Table S3.** The effect of medication on ICA findings in the BD and MDD group (MDD - Major Depressive Disorder, BD – Bipolar Disorder, ICA – Independent Component Analysis, IC – Independent Component, SSRI - Selective Serotonin Reuptake Inhibitors, DMN – Default Mode Network, SN – Salience Network, FPN – Frontoparietal Network, SMN – Sensorimotor Network).

| **Patient Group** | **Component** |  | **Regions** | ***P* Value** | | ***T* Score** | |  |  |
| --- | --- | --- | --- | --- | --- | --- | --- | --- | --- |
| MDD SSRI vs MDD not currently on SSRI | 4 (DMN)  9 (SN)  10 (FPN)  12 (SN)  18 (DMN)  31 (SMN) |  | Angular Gyrus  Inferior Temporal Gyrus  Lateral Occipital Cortex  Lateral Occipital Cortex  Postcentral Gyrus  Lateral Occipital Cortex  Inferior Temporal Gyrus  Inferior Frontal Gyrus  Intracalcarine Cortex  Occipital Fusiform Gyrus | .630  .504  .110  .399  .021  .594  .205  .919  .845  .489 | -.485  -.675  -1.639  .854  -2.415  -.538  -1.290  -.103  .197  -.699 | |  | | |

| **Patient Group** | **Component** |  | **Regions** | ***P* Value** | ***T* Score** | |  |  |
| --- | --- | --- | --- | --- | --- | --- | --- | --- |
| BD Lithium vs BD not currently on Lithium | 4 (DMN)  9 (SN)  10 (FPN)  12 (SN)  18 (DMN)  31 (SMN) |  | Angular Gyrus  Inferior Temporal Gyrus  Lateral Occipital Cortex  Lateral Occipital Cortex  Postcentral Gyrus  Lateral Occipital Cortex  Inferior Temporal Gyrus  Inferior Frontal Gyrus  Intracalcarine Cortex  Occipital Fusiform Gyrus | .308  .208  .114  .631  .075  .205  .464  .850  .828  .665 | -1.033  -1.283  -1.620  .485  1.835  -1.291  -.740  .190  -.219  -.436 |  | | |

**S4. Spatial to template Neural Network matching for ICA analysis**

This figure illustrates the method of how the 32 independent components in our dataset were matched to neural networks. Three correlation coefficient values were provided for each component indicating which three networks were most common within the component and the network with the highest coefficient value was assigned to the component.


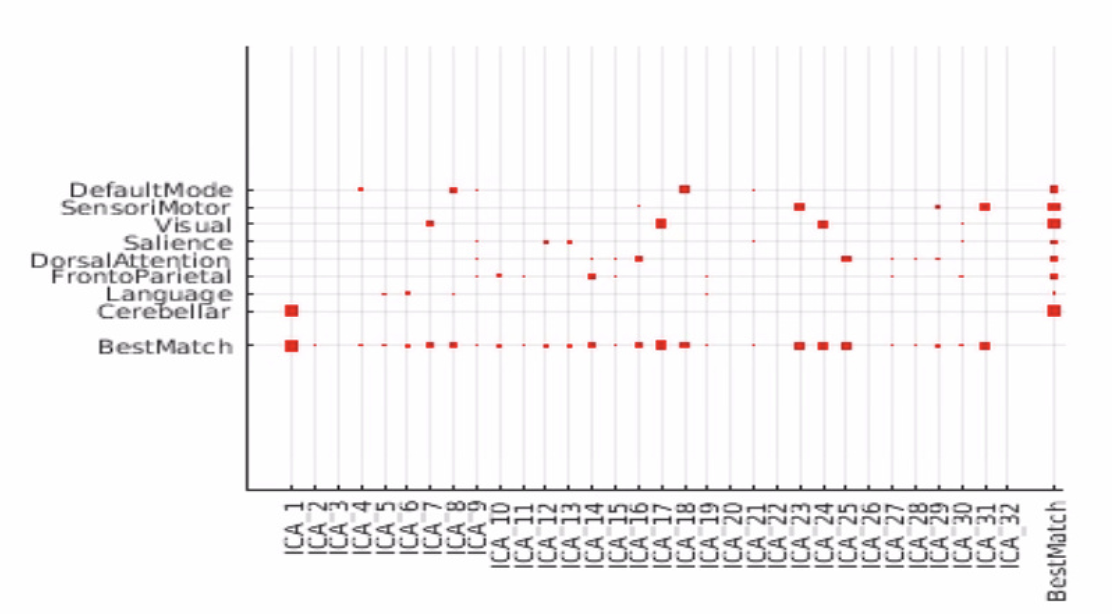


**Figure S2.** The spatial matching template generated by CONN representing which of the extracted components from the dataset best match each resting state network.

**S5. Comparisons between Patient Groups and Healthy Controls from ICA analysis.**

A one-way ANOVA with a threshold of *p* < .05 was conducted to investigate differences between BD and MDD relative to HC across the significant connectivity findings of the ICA analysis. Following this we adjusted for multiple comparisons using the Bonferroni correction method to determine if there were differences between MDD or BD groups versus the HC group. The post-hoc results are summarised below for MDD vs HC groups in Table S4 and for BD vs HC groups in Table S5.

**Table S4.** Differences in ICA connectivity at rest for MDD vs HC.

| **Component** | **Neural Network** | ***F* Score** | **Contrast** | **Regions** | ***p* Value (<.05)** |
| --- | --- | --- | --- | --- | --- |
| 4 | DMN | 12. 947  13. 709  11.328 | MDD > HC  MDD > HC  MDD > HC | Angular Gyrus  Inferior Temporal Gyrus  Lateral Occipital Cortex | .055  .001  .001 |
| 9 | SN | 15.768 | MDD > HC | Lateral Occipital Cortex | .001 |
| 10 | FPN | 14.265 | MDD > HC | Postcentral Gyrus | .075 |
| 12 | SN | 10.473 | MDD > HC | Lateral Occipital Cortex | .043 |
| 18 | DMN | 11.168  11.145 | MDD > HC  MDD > HC | Inferior Temporal Gyrus  Inferior Frontal Gyrus | .211  .211 |
| 31 | SMN | 8.763  9.757 | MDD > HC  MDD > HC | Intracalcarine Cortex  Occipital Fusiform Gyrus | .138  .084 |

**Table S5.** Differences in ICA connectivity at rest for BD vs HC.

| **Component** | **Neural Network** | ***F* Score** | **Contrast** | **Regions** | ***p* Value (<.05)** |
| --- | --- | --- | --- | --- | --- |
| 4 | DMN | 12. 947  13. 709  11.328 | BD > HC  BD > HC  BD > HC | Angular Gyrus  Inferior Temporal Gyrus  Lateral Occipital Cortex | .024  .387  1.00 |
| 9 | SN | 15.768 | BD > HC | Lateral Occipital Cortex | .244 |
| 10 | FPN | 14.265 | BD > HC | Postcentral Gyrus | .008 |
| 12 | SN | 10.473 | BD > HC | Lateral Occipital Cortex | .115 |
| 18 | DMN | 11.168  11.145 | BD > HC  BD > HC | Inferior Temporal Gyrus  Inferior Frontal Gyrus | .014  .015 |
| 31 | SMN | 8.763  9.757 | BD > HC  BD > HC | Intracalcarine Cortex  Occipital Fusiform Gyrus | .093  .088 |
